# Functional morphology of antennae and sensilla of the fungivore beetle, *Triplax ainonia* Lewis (Coleoptera: Erotylidae)

**DOI:** 10.1371/journal.pone.0309670

**Published:** 2024-08-29

**Authors:** Xiao-Long Hou, Shi-Hui Huang, Ben Hong, Mao-Fa Yang, Chang-Qing Luo

**Affiliations:** 1 Guizhou Provincial Key Laboratory for Agricultural Pest Management of the Mountainous Region, Institute of Entomology, Guizhou University, Guiyang, PR China; 2 College of Animal Science of Guizhou University, Guizhou University, Guiyang, PR China; 3 College of Tobacco Science of Guizhou University, Guizhou University, Guiyang, PR China; Beni Suef University Faculty of Veterinary Medicine, EGYPT

## Abstract

The antennal sensilla play an important role in many behavioral activities of insects. The fungivorous beetle *Triplax ainonia* Lewis (Erotylidae) is an important pest which prefers to feed on *Pleurotus* mushrooms. In order to clarify the types, number, and distribution of the antennal sensilla of male and female *T*. *ainonia*, scanning electron microscopy was used. The results showed that there were five sensillum types on the antennae of adults male and female, including Böhm’s bristles (BB), sensilla chaetica (three subtypes: SC 1, SC 2, and SC 3), sensilla basiconica (three subtypes: SB 1, SB 2, and SB 3), sensilla trichodea (ST), and sensilla styloconica (SS). Among all the sensilla, the number of SB 2 was the most abundant in both sexes. We found that there was no sexually dimorphic in the sensillum types, but there were differences in the number, lengths, and diameters of some sensilla between males and females. Based on the information of the morphology and distribution of the sensilla, the potential functions of the antennal sensilla of *T*. *ainonia* adults were discussed. The results of this study provide a basis for further study on the behavioral ecology and electrophysiology of the fungivore beetles of the Erotylidae.

## Introduction

Erotylidae is one of the major families of Coleoptera, with about 136 genera and 3,000 species worldwide [1]. The family is also known as “Pleasing fungus beetles” because of its diverse shape, bright color, and fungus-feeding behavior [2]. Beetles of the genus *Triplax* often feeds on *Pleurotus* mushrooms, which represent as an important pest of edible fungi [2]. The species *Triplax ainonia* Lewis belongs to the genus *Triplax* Herbst of the tribe Tritomini of the subfamily Erotylinae, which are mainly distributed in China, Japan, and South Korea [3, 4]. *Triplax ainonia* Lewis is similar to most other species of the genus *Triplax* (e.g. *T*. *aenae* Schaller and *T*. *rufipes* Fabricius) [5, 6], prefers to feed on the mushrooms of the genus *Pleurotus* (e.g. *P*. *ostreatus*). The oyster mushroom, *P*. *ostreatus* (Jacq.) Kumm is the second most cultivated edible mushroom worldwide after the button mushroom *Agaricus bisporus* (J.E. Lange) Imbach. It has economic and ecological values in addition to medicinal properties [7]. Both larvae and adults of *T*. *ainonia* can feed on the fruiting bodies of *P*. *ostreatus*. In the environmental conditions of high-temperature and humidity, *T*. *ainonia* will cluster outbreak, and usually hidden in the fruiting body or between the gills of the mushroom, and the excrement of insects will pollute the mushroom body, thus affecting the quality of edible fungi. In severe cases, the whole fruiting body of *P*. *ostreatus* can be eaten into powdery by *T*. *ainonia*, and resulting in a serious decline in mushroom production, or even near-total crop failure.

The antennae are one of the important components of the sensory system of insects, such as foraging, nesting, mating, migration, and oviposition [8–11]. Many types of sensillum are usually distributed on the antennae of insects, including Böhm’s bristles (BB), sensilla trichodea (ST), sensilla chaetica (SC), sensilla basiconica (SB), and sensilla styloconica (SS), etc. [12–14]. The antennal sensilla have mechanical perception, chemical perception, and thermo-hygroreceptive functions [15–17]. For example, the external morphology of BB, ST, and long SC is consistent with the overall characteristics of the mechanosensory sensilla, and these sensilla usually have tactile sensing functions [18–20]. In addition, the SC have chemosensory function, which characterized by the terminal hole at the blunt tip of the sensillum, and thus the SC are presumed to have taste function [21]. The SB on the antennae of insects is a common sensilla with chemical perception function, and plays a key role in the process of receiving host odors [22]. The SS are common in Lepidopterous insects and have been shown to have the thermo-hygroreceptive function [23–26]. Moreover, the SS were also found on the antennae of Coleoptera insects, and electrophysiological studies confirm similar function as in Lepidopteran [27, 28].

Up to date, there have been a large number of morphological studies on species of Erotylidae, and the ultramorphology of various structures, such as the antennae, mouthparts, eyes, legs, elytra, glands, and genitalia has also been reported [29–32]. The antennae of species of the family Erotylidae are composed of 11 segments [15, 33]. The scape is stout, and the pedicel is approximately circular. The flagellum consists of 9 flagellomeres, and the 3–5 flagellomeres at the terminal are expanded laterally, forming an abrupt, flat, oval club [29, 33]. However, the studies on the beetle *T*. *ainonia* focused mainly on its classification and geographical distribution [33, 34]. The ultrastructure of the antennal sensilla of Erotylidae insects has been little studied, and there are also no reports on the ultrastructure of the antennal sensilla of the beetle *T*. *ainonia*.

In the present study, the types, number, and distribution of antennal sensilla of adults male and female of *T*. *ainonia* were investigated using scanning electron microscopy (SEM). Such results will be useful for further studies on host-seeking mechanisms, electrophysiology, and behavioral ecology for *T*. *ainonia*, which can contribute to the effective control of this pest.

## Materials and methods

### Ethics statement

The beetle *T*. *ainonia* used in the present study is not classified as an endangered or protected species, and is also not included in the “List of Protected Animals in China”. This beetle species is a serious pest of cultivated mushroom *P*. *ostreatus* in China, and all insect samples were collected from agricultural land for the purpose of commercial mushroom production. Therefore, no specific permits were required for this beetle species and to conduct sampling in the location referred to in this study.

### Insects

*Triplax ainonia* Lewis were collected from the farm of the oyster mushroom, *P*. *ostreatus* in Baijin Town, Huishui County, Qiannan Autonomous Prefecture, Guizhou Province (26°6′18.7776″N, 106°49′26.0508″E). The collected adults of *T*. *ainonia* (male: N = 20; female: N = 20) were placed in a cage (120 cm×60 cm×40 cm) and fed with fresh *P*. *ostreatus* in a climate chamber with a temperature of (25±1)°C, relative humidity of (70±5)%, and light period of L:D = 16 h:8 h.

### Scanning electron microscopy

The heads of *T*. *ainonia* adults (male: N = 20; female: N = 20) were dissected using microscissors under a stereomicroscope (Nikon SMZ1270, Nikon Corporation, Japan). According to the method of Zhu et al. [35], the antennae immediately fixed in glutaraldehyde (2.5%) at 4°C for 20 h, and then washed with phosphate-buffer solution (0.1 mol/L, PH = 7.4) three times, each time for 15 min. Dehydrat ion process was performed using graded alcohol series (75%, 80%, 85%, 90%, 95%, and 100%) for 15 minutes per each concentraion, and finally fixed them in tert-Butyl alcohol (99%) at 37°C. Subsequently, drying for 12 hours at 40°C in an electric blast dryer (101-ISB, Shaoxing Supo Instrument Co., Ltd.) was performed. The antennae were then fixed to metallic stubs with double-sided carbon tape and coated with gold film for 2 minutes with an ion sputtering instrument (NeoCoater, Hitachi, Japan). The antennae were investigated under at an accelerating voltage of 15 kV scanning electron microscope (JCM 6000, Hitachi, Japan).

The nomenclature used to describe the sensillum types according to previous scientific literatures on Coleopteron insects [9, 10, 20, 27, 36]. The number of various sensillum types were counted to reflect their distribution on the whole antenna. The sizes (lengths and diameters) of various sensillum types were measured using the measurement tool of SEM.

### Analysis

The mean length and width of each antennal segment (N = 10), as well as the number, distribution, lengths, and base diameters of each sensillum type (N = 20) were measured and counted. The statistical analysis was conducted using the software SPSS 27.0 (IBM, Armonk, USA), and the obtained data were described by mean ± standard error. If the measured data conforms to the normal distribution and homogeneity of variance, the independent sample *T* test is used to determine whether there is a significant difference between males and females. Conversely, the *U* test was used. All significant difference levels were set to 0.05. The software Origin 2022 (OriginLab Corp, USA) was used to visualize the data.

## Result

### Morphological characteristics of adult antennae

**Fig 1:**
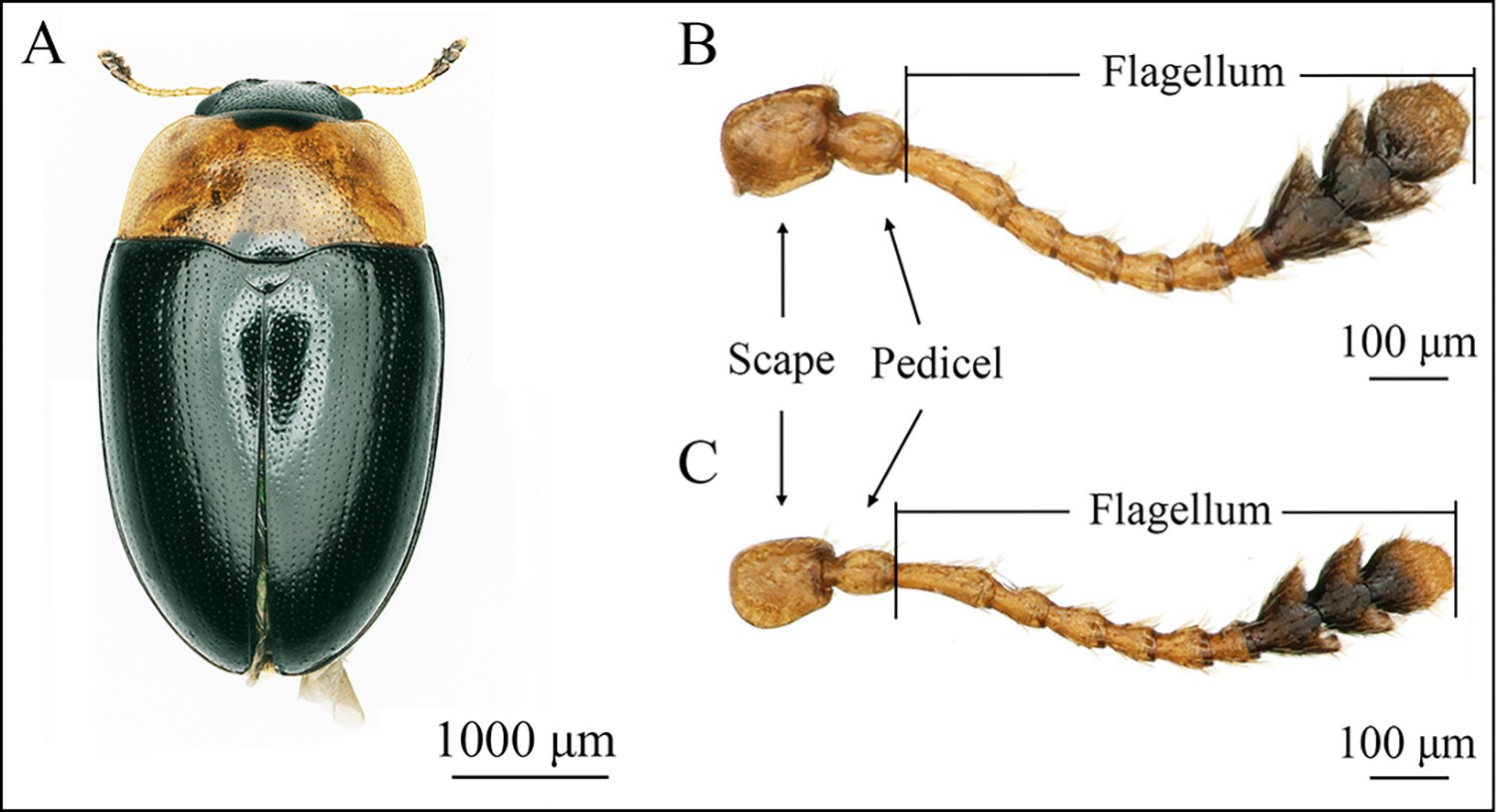
The morphology of the antennae of *T*. *ainonia* adults. (A) *T*. *ainonia* adult; (B) Antennae of male; (C) Antennae of female.

**Fig 2:**
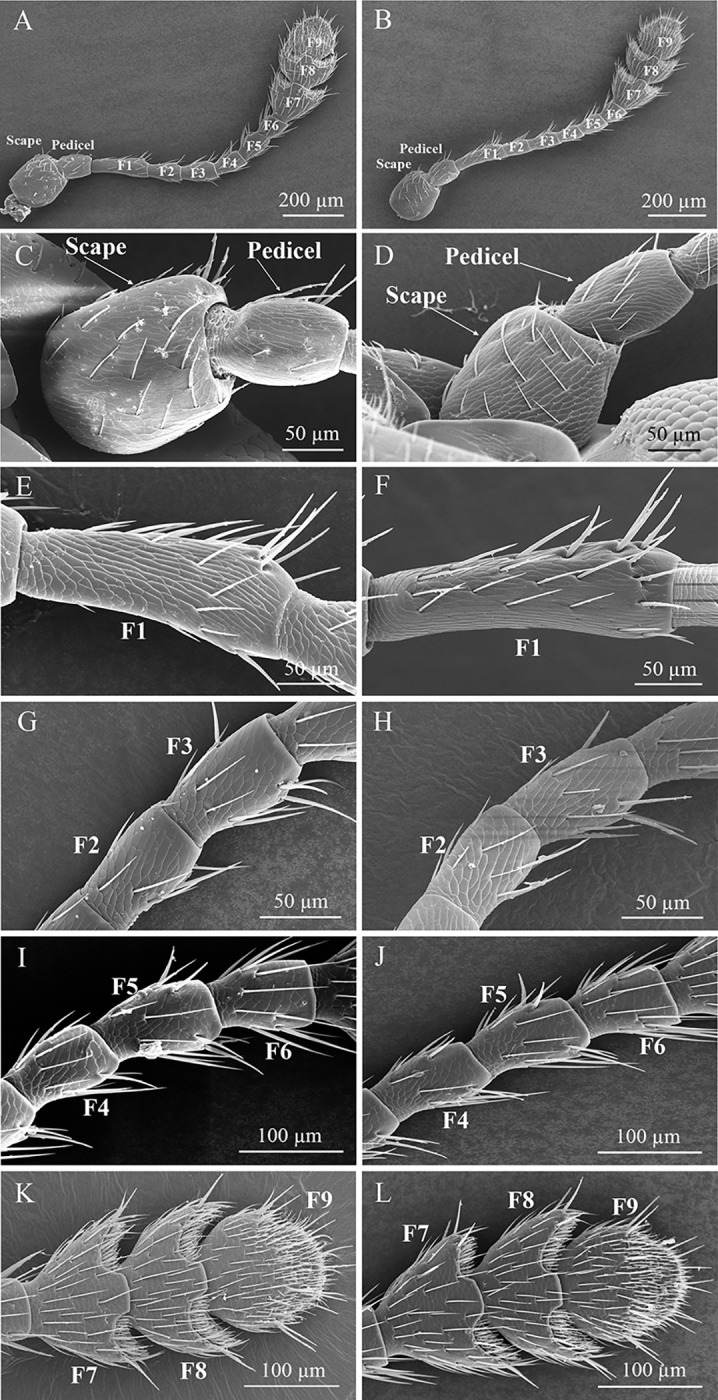
Ultrastructure of antennal segment of *T*. *ainonia* adults. (A) Antennae of male; (B) Antennae of female; (C) Scape and pedicel of male; (D) Scape and pedicel of female; (E) 1st flagellomere (F1) of male; (F) 1st flagellomere (F1) of female; (G) 2nd and 3rd flagellomeres (F2 and F3) of male; (H) 2nd and 3rd flagellomeres (F2 and F3) of female; (I) 4th–6th flagellomeres (F4–F6) of male; (J) 4th–6th flagellomeres (F4–F6) of female; (K) 7th–9th flagellomeres (F7–F9) of male; (L) 7th–9th flagellomeres (F7–F9) of female.

The antennae of adults male and female of *T*. *ainonia* are composed of scape, pedicel and flagellum (Figs 1; 2A and 2B). The scape is cylindrical and stout, while the pedicel is oval, and significantly narrower than the scape (Fig 2C and 2D). The flagellum consists of 9 flagellomeres (F1–F9) (Fig 2E–2L), and the F1 is the longest flagellomere (Fig 2E and 2F). The shape of F2–F6 is similar as a short cylinder (Fig 2G–2J). The shape of F7, F8, and F9 were nearly triangular, semilunar, and circular, respectively (Fig 2K and 2L).

### Lengths and widths of antennal segments

**Fig 3:**
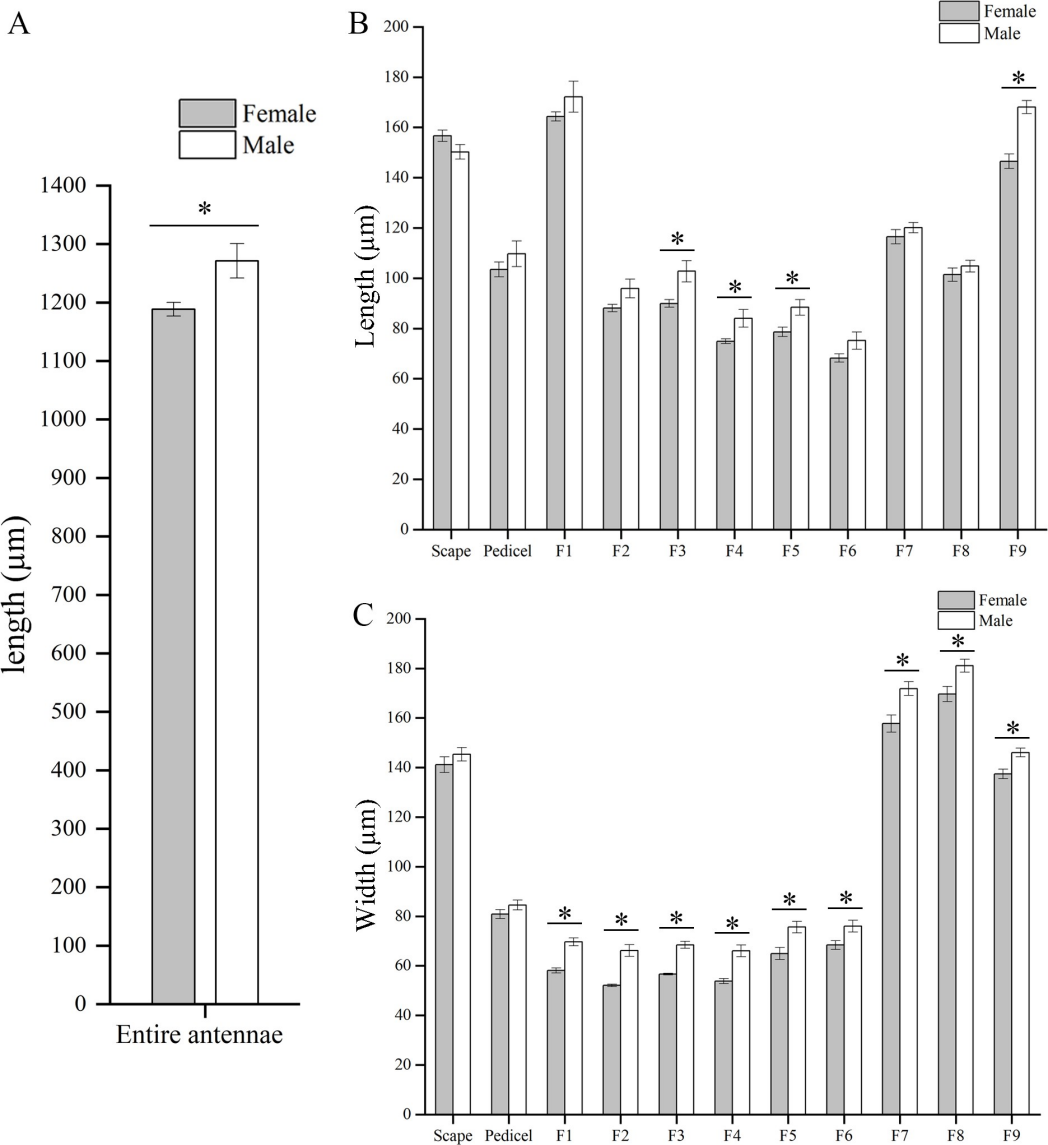
The lengths and widths of each antennal segment for both sexes of *T*. *ainonia*. (A) The length of entire antennae of *T*. *ainonia*; (B) The length of each antennal segment. (C) The width of each antennal segment. Asterisk indicates significant difference in 0.05 level.

Details of the lengths and widths of different antennal segments are shown in Fig 3. The entire antennal morphology of adults male and female of the *T*. *ainonia* is consistent, but the total length of the antennae of male adults is longer than that of female adults (*P* < 0.05; Fig 3A). The lengths of F3–F5 and F9 of flagellum of males are significantly longer than that of females (*P* < 0.05), while there are no significant differences in the lengths of scape, pedicel, and five flagellomeres of flagellum (F1, F2, F6, F7, and F8) between male and female adults of *T*. *ainonia* (*P* > 0.05; Fig 3B). The scape, F7, F8, and F9 of the antennae of adults male and female are thicker compared to other antennal segments (Fig 3B and 3C). The difference in the width of the antennal segments between male and female adults is mainly reflected in the flagellum. The width of all flagellomeres of male flagellum is significantly wider than females (*P* < 0.05). However, no significant difference in the width of antennal scape and pedicel between the two sexes was recorded (*P* > 0.05; Fig 3C). The slenderest antennal segment of adults male and female is F1. The lengths of F1 of the female are about 2.83 times the width, while measure 2.47 times for male. The shortest antennal segment of adults male and female is F6, which is approximately equal in length and width.

### Distribution of the antennal sensilla

**Fig 4:**
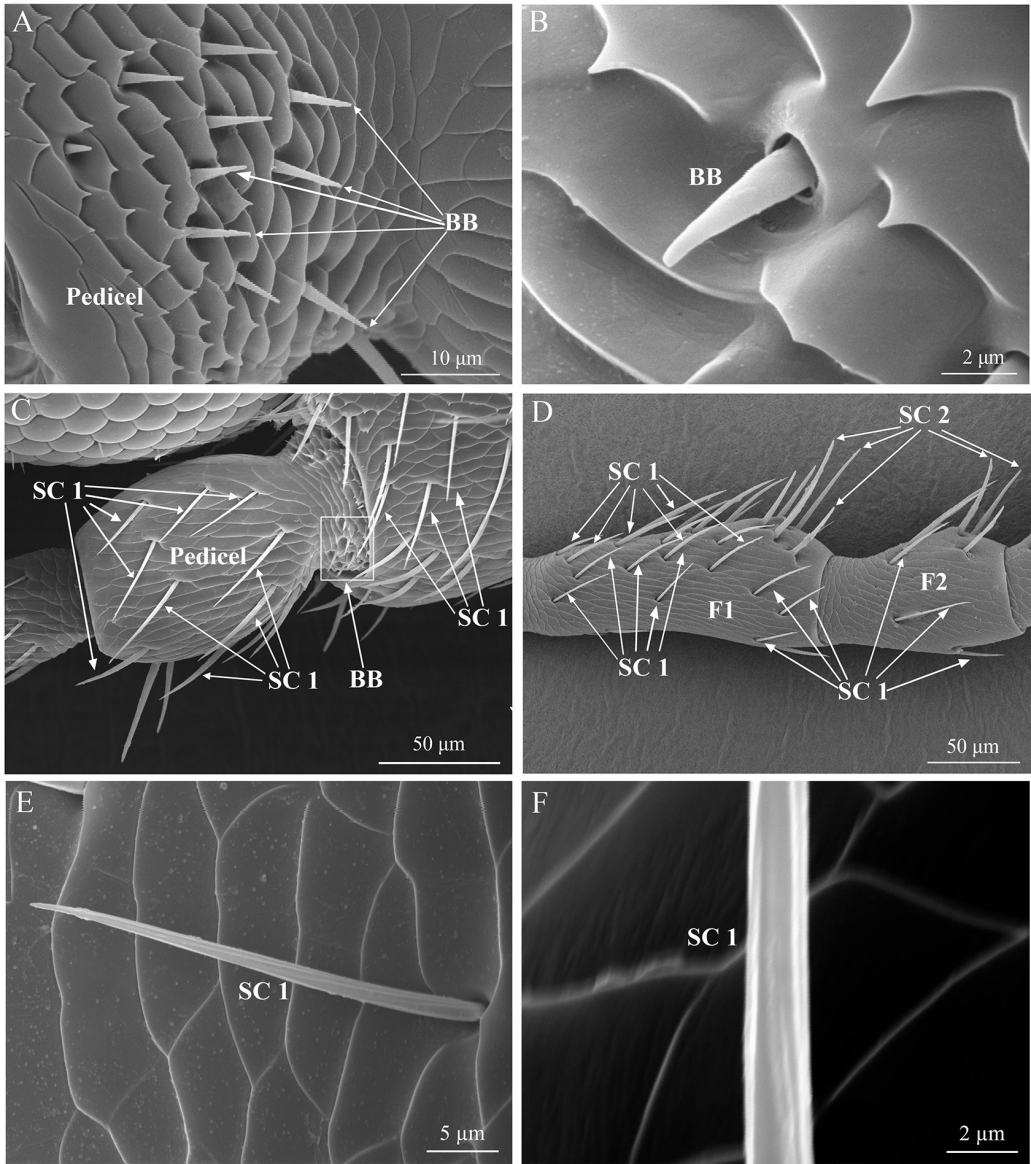
Micrographs of scape, pedicel, and 2 flagellomeres of *T*. *ainonia*. (A), (B) Böhm’s bristles (BB); (C) BB and SC 1; (D) Distribution of different sensillum types on the 1st and 2nd flagellomeres (F1 and F2): SC 1 and SC 2; (E) The overall view of SC 1; (F) Magnified view of the grooved wall of SC 1.

**Fig 5:**
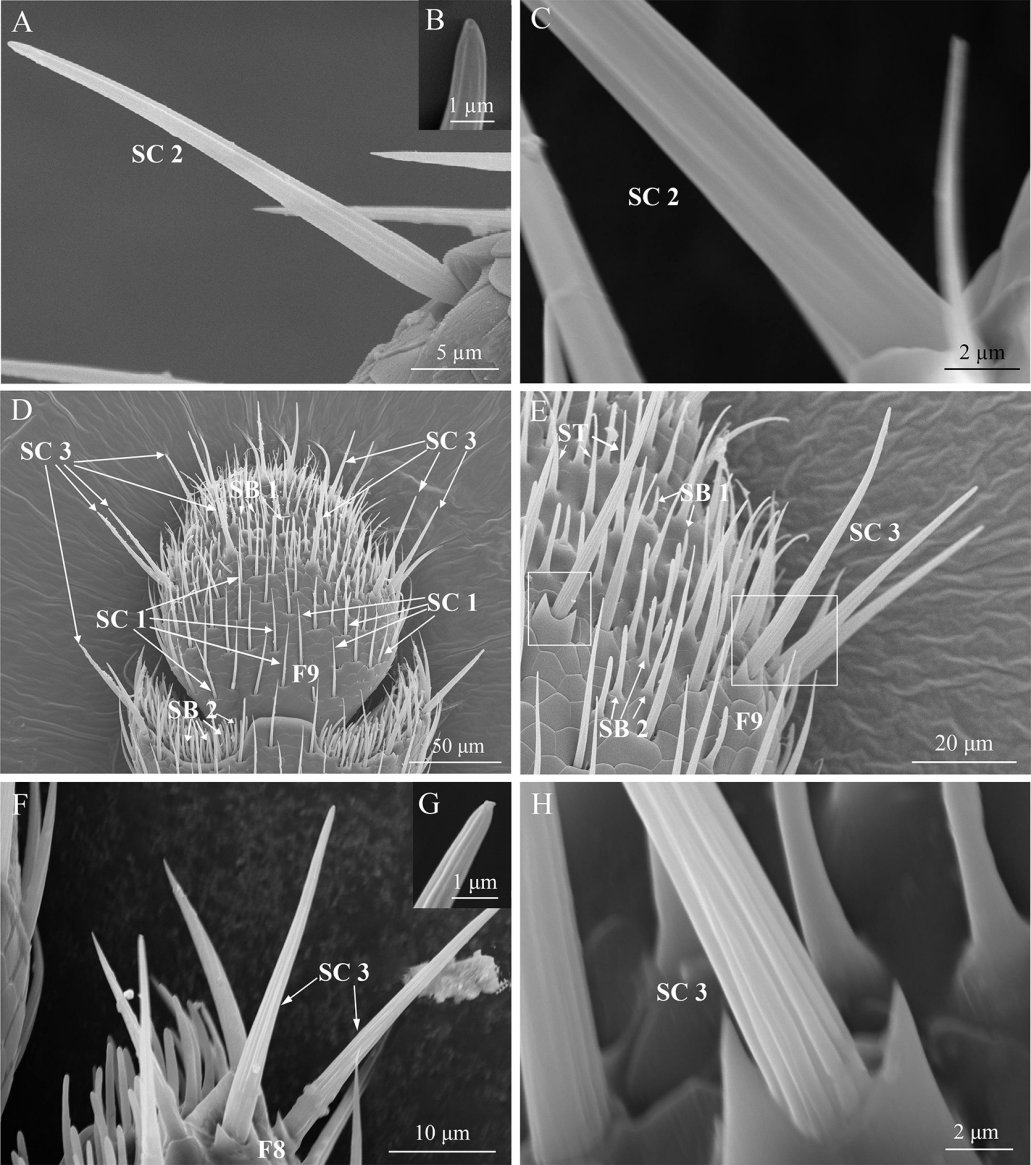
Micrographs of different subtypes of sensilla chaetica. (A) The overall view of SC 2; (B) Magnified view of the terminal end of SC 2; (C) Magnified view of the grooved wall of SC 2; (D), (E) Distribution of different sensillum types on the 8th and 9th flagellomeres (F8 and F9): SC 1, SC 3, SB 1, SB 2, and ST; (F) The overall view of SC 3; (G) Magnified view of the terminal end of SC 3; (H) Magnified view of the grooved wall of SC 3.

**Fig 6:**
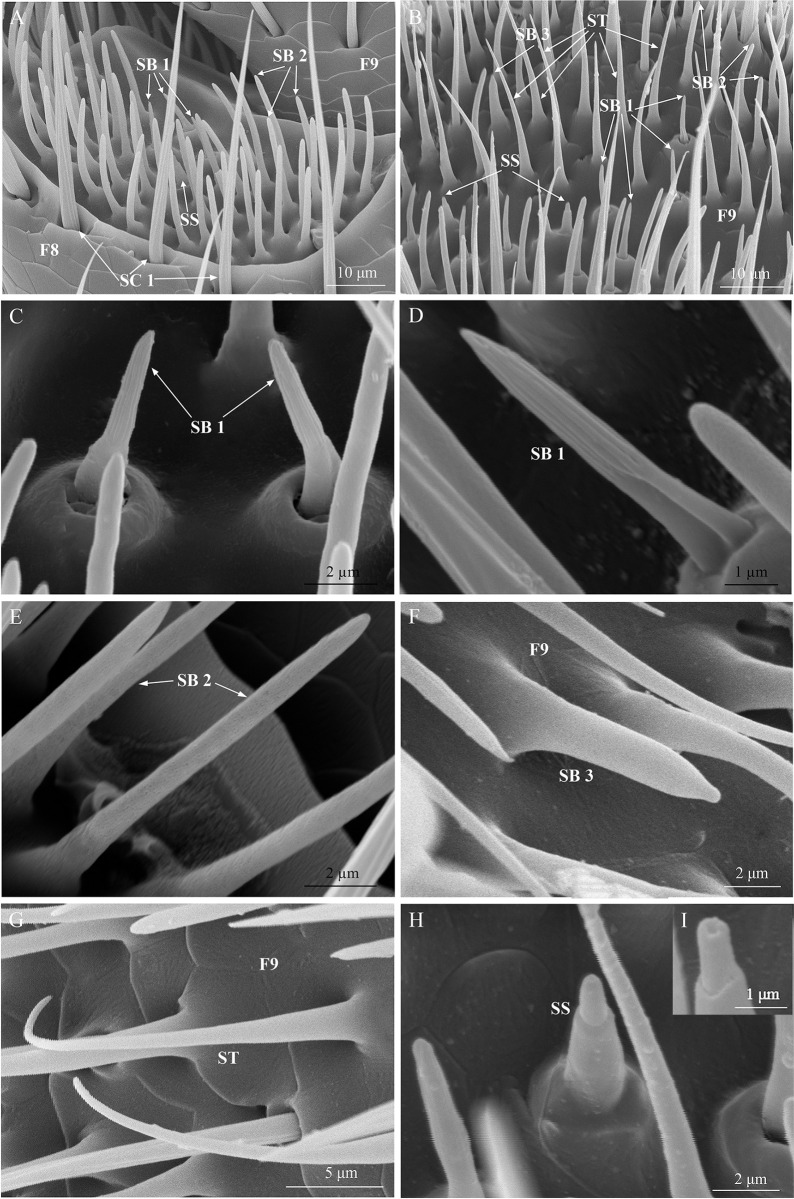
Micrographs of various sensillum types on flagellum segments. (A) Distribution of different sensillum types on the 8th flagellomere (F8): SC 1, SB 1, SB 2, and SS; (B) Distribution of different sensillum types on the 9th flagellomere (F9): SB 1, SB 2, SB 3, ST, and SS; (C) The overall view of SB 1; (D) Magnified view of SB 1; (E) The overall view of SB 2; (F) The overall view of SB 3; (G) The overall view of ST; (H) The overall view of SS; (I) Magnified view of the terminal end of SS.

There are five sensillum types distributed on the antennae of both sex (male and female) adults of *T*. *ainonia*: Böhm’s bristles (BB), sensilla trichodea (ST), sensilla chaetica (SC), sensilla basiconica (SB), and sensilla styloconica (SS) (Table 1 and Figs 4–6). According to the external morphological characteristics and size of the sensilla, the sensilla chaetica and sensilla basiconica are further divided into three subtypes, respectively (Table 2).

**Table 1:** The distribution and number of various sensillum types on the antennal segments of adult *T*. *ainonia*.

Sensillum type	Sex	Number of sensilla										
		Scape	Pedicel	Flagellum								
				F1	F2	F3	F4	F5	F6	F7	F8	F9
Böhm’s bristles	Female	–	13.65±0.34 a	–	–	–	–	–	–	–	–	–
	Male	–	13.95±0.27 a	–	–	–	–	–	–	–	–	–
Sensilla chaetica subtype 1	Female	24.20±0.68 b	9.15±0.29 b	16.30±0.59 a	7.80±0.35 a	7.30±0.27 b	8.85±0.33 a	9.10±0.33 b	10.65±0.27 b	34.65±0.93 b	40.00±0.95 b	48.95±1.05 b
	Male	26.80±0.68 a	10.85±0.37 a	16.40±0.94 a	7.70±0.31 a	8.50±0.41 a	7.95±0.29 a	10.85±0.42 a	11.75±0.25 a	43.05±0.82 a	49.80±0.96 a	55.65±1.09 a
Sensilla chaetica subtype 2	Female	2.60±0.15 a	2.00±0.15 a	3.45±0.15 b	0.70±0.16 b	3.65±0.13 a	–	3.60±0.17 a	–	–	–	–
	Male	2.80±0.19 a	2.00±0.10 a	4.90±0.28 a	2.55±0.17 a	3.20±0.17 b	2.15±0.22	3.90±0.18 a	–	–	–	–
Sensilla chaetica subtype 3	Female	–	–	–	–	–	–	–	–	5.60±0.17 a	5.70±0.15 a	15.90±0.32 a
	Male	–	–	–	–	–	–	–	–	5.90±0.16 a	6.05±0.15 a	15.85±0.32 a
Sensilla basiconica subtype 1	Female	–	–	–	–	–	–	–	–	4.35±0.24 a	6.4±0.4 a	9.80±0.43 a
	Male	–	–	–	–	–	–	–	–	4.50±0.28 a	6.65±0.33 a	9.65±0.49 a
Sensilla basiconica subtype 2	Female	–	–	–	–	–	–	–	–	83.05±1.4 a	105.55±1.97 a	34.40±1.07 a
	Male	–	–	–	–	–	–	–	–	83.35±2.19 a	105.3±2.21 a	35.00±1.38 a
Sensilla basiconica subtype 3	Female	–	–	–	–	–	–	–	–	–	–	1.10±0.07 a
	Male	–	–	–	–	–	–	–	–	–	–	1.25±0.12 a
Sensilla trichodea	Female	–	–	–	–	–	–	–	–	–	–	33.15±0.87 a
	Male	–	–	–	–	–	–	–	–	–	–	31.85±1.13 a
Sensilla styloconica	Female	–	–	–	–	–	–	–	–	0.75±0.14 a	1.30±0.23 a	2.25±0.19 a
	Male	–	–	–	–	–	–	–	–	0.20±0.09 b	0.55±0.15 b	1.90±0.18 a

Data were expressed as mean ± SE (N = 20). The data of the same type of sensillum are followed by lowercase letters. These letters indicate whether the number of sensilla of the same subtype is significantly different between the sexes in the same antenna segment (The same letter indicates that the difference is not significant, and different letters indicate that the difference is significant) (independent-samples *T*-test; *P* < 0.05). “–” indicates that no sensilla are observed.

**Table 2:** The lengths and basal diameters of different sensillum types on antennae of adults *T*. *ainonia*.

Sensillum type	Sex	Length/μm	Basal diameter/μm
Böhm’s bristles	Male	5.58±0.26 a	1.32±0.02 a
	Female	5.73±0.18 a	1.32±0.02 a
Sensilla chaetica subtype 1	Male	44.56±2.18 a	2.58±0.14 a
	Female	42.11±1.26 a	2.24±0.12 a
Sensilla chaetica subtype 2	Male	43.32±0.96 a	3.75±0.10 a
	Female	45.57±1.32 a	3.51±0.11 a
Sensilla chaetica subtype 3	Male	49.41±1.35 a	3.83±0.12 a
	Female	52.32±1.89 a	3.67±0.08 a
Sensilla basiconica subtype 1	Male	7.71±0.14 a	1.52±0.05 a
	Female	7.77±0.16 a	1.43±0.07 a
Sensilla basiconica subtype 2	Male	15.46±0.29 b	2.65±0.06 a
	Female	16.49±0.39 a	2.34±0.06 b
Sensilla basiconica subtype 3	Male	7.73±0.21a	2.64±0.08 a
	Female	6.85±0.22 b	2.58±0.06 a
Sensilla trichodea	Male	21.12±0.47 a	2.37±0.04 a
	Female	19.70±0.43 b	2.39±0.06 a
Sensilla styloconica	Male	5.08±0.08 a	2.52±0.09 a
	Female	4.26±0.10 b	2.38±0.09 a

Data were expressed as mean ± SE (N = 20). The data of the same subtype of sensillum is followed by lowercase letters. These letters indicate whether there is a significant sexes difference between the same statistics, (the same letter indicates that the difference is not significant, and the different letters indicate that the difference is significant) (independent sample *T*-test; *P* < 0.05).

#### Böhm’s bristles

Böhm’s bristles (BB) are bristly, upright, and situated in a mortar-shaped cuticular sockets (Fig 4A and 4B). There are no pores on the surface of this sensillum (Fig 4B). These sensilla are distributed on the antennal pedicel (at the junction with the scape) of both sexes (Fig 4C and Table 1). There is no significant difference in the number, lengths, and basal diameters of BB sensillum between the both sexes (*P* > 0.05; Tables 1 and 2). Moreover, rows of microtrichia are found on the base of the pedicel (Fig 4A and 4B).

#### Sensilla chaetica

There are three subtypes of sensilla chaetica on the antennae of *T*. *ainonia*: SC 1, SC 2, and SC 3. The shape of SC 1 is thorn-like, with a socket at the base, and an angle between these sensilla and the antenna surface is less than 45° (Fig 4C–4E). In addition, there are longitudinal grooves on the surface of SC 1, and the its tip is sharp (Fig 4E and 4F). These sensilla are distributed on each segment of antennae (Table 1). There is no significant difference between the two sexes in the number of SC 1 in F1, F2, and F4 of Flagellum (*P* > 0.05), but the number of SC 1 in other flagellomeres of males is significantly higher than female (*P* < 0.05; Table 1). There is no significant difference between males and females in lengths and base diameters (*P* > 0.05; Table 2).

The SC 2 are bristled, grooved wall, and slightly curved toward the outside of the antennal surface (Fig 5A and 5C). The position near the base of SC 2 is enlarged, and the enlargement gradually becomes thinner from the base to tip. The base has a socket, and its tip is blunt with a terminal pore (Fig 5B and 5C). The SC 2 are distributed on the antennal scape, pedicel, and F1 to F5 of flagellum (except for the F4 of females) (Table 1). There is no significant difference in the number of SC 2 on scape, pedicel, and F5 of flagellum between both sexes (*P* > 0.05; Table 1). The number of SC 2 on F1 and F2 flagellomeres of males is significantly higher than that in females (*P* < 0.05; Table 1). The number of SC 2 on F3 is significantly higher in females (*P* < 0.05; Table 1). The lengths and basal diameters of SC 2 between males and females don’t differ significant (*P* > 0.05; Table 2).

The external morphology of SC 3 is similar to SC 2, but longer in their lengths (range: 49.41–52.32 μm) than SC 2 (range: 42.11–43.32 μm) in both sexes (Fig 5D–5F). There is a disc-shaped pore at its terminal end (Fig 5G). The base of these sensilla has a socket, and there are two small triangular shields on both sides of the sockets. The base of SC 3 is embedded in the groove between the two shields (Fig 5F and 5H). The SC 3 are distributed at the ends of both sides of the F7 to F9 flagellomeres (Fig 5D and Table 1). The number of SC 3 distributed in each segment between males and females don’t show significant differences (*P* > 0.05; Table 1). Moreover, no significant difference in lengths and base diameters between males and females was recorded (*P* > 0.05; Table 2).

#### Sensilla basiconica

Sensilla basiconica are divided into three subtypes (SB 1, SB 2, and SB 3). The SB 1 are a double-walled basiconic sensilla, characterized by a short, fluted cuticular surface with a grooved peg-like appearance distally (Fig 6A–6D). The base of these sensilla has a socket with cotyloid bulge (Fig 6C). The SB 1 are distributed on the F7–F9 of flagellum. The SB 1 are distributed in the grooves at the end of F7 and F8 (Fig 6A), and in F9, mainly distributed on the upper half part (Fig 6B). The number, lengths, and base diameters of SB 1 between the two sexes don’t differ significant (*P* > 0.05; Tables 1 and 2).

The SB 2 are finger-like, slightly curved in shape. The base of the SB 2 has no socket, but bulge. The surface of these sensilla is porous, with obtuse tip (Fig 6E). The SB 2 are abundantly distributed on F7–F9 of flagellum. The distribution pattern of SB 2 on F7 and F8 is similar to SB 1 (Fig 6A), While it is distributed on the middle part of 9th flagellomere (Fig 6B). There is no significant difference in the number of SB 2 between both sexes (*P* > 0.05; Table 1). On the other hand, the lengths of the SB 2 in females are significantly longer than that of males (*P* < 0.05), and the base diameters of the SB 2 in males are significantly larger than female (*P* < 0.05; Table 2).

There are no sockets at the base of the SB 3 and it’s obviously enlarged, upright. These sensilla are characterized by a smooth wall without longitudinal grooves, a uniform thickness, and a distinctive droplet shape at the tip (Fig 6F). The SB 3 only distributed on the middle and upper part of 9th flagellomere (Fig 6B). There is no significant difference in the number of SB 3 between both sexes (*P* > 0.05; Table 1). The lengths of the sensilla in males are significantly longer than females (*P* < 0.05; Table 2), While the widths of SB 3 between both sexes don’t differ significant (*P* > 0.05; Table 2).

#### Sensilla trichodea

There is only one type of sensilla trichodea (ST) was recorded in the antennal segments. The shape of the ST is hair-like, with no sockets at the base, curved and sharp tip. The surface of the ST is smooth-wall, without grooves or pores (Fig 6G). ST sensilla are distributed only on F9 (Fig 6B and Table 1). The number, lengths, and base diameters of ST don’t show any significant difference between both sexes (*P* > 0.05; Tables 1 and 2).

#### Sensilla styloconica

Sensilla styloconica (SS) have no sockets at the base of the SS (Fig 6H). The surface of SS is smooth-wall, and the tip is obtuse and has a pore (Fig 6I). The SS are distributed on the terminal flagellomeres F7–F9. These sensilla are found in the grooves at the end of the F7 and F8, and on the middle and terminal part of the F9 in both sexes (Fig 6A and 6B). The number of the SS is significantly higher in females than males (*P* < 0.05; Table 1). The lengths of the SS in males are significantly longer compared to females (*P* < 0.05), whereas, there is no significant difference in the widths of the SS between the two sexes (*P* > 0.05; Table 2).

## Discussion

In this study, we identified five types of sensilla on the antennae of *T*. *ainonia* beetle. The Böhm’s bristles (BB), sensilla trichodea (ST), sensilla chaetica (SC), and sensilla basiconica (SB) found in *T*. *ainonia* were also ubiquitous in other Coleoptera insects [16–18, 37–39]. However, the sensilla styloconica (SS) of *T*. *ainonia* were relatively less reported in Coleoptera insects [27, 28]. Although there are many reports on the morphology of the species belonging to the family Erotylidae, most studies have not involved the ultrastructure of the antennal sensilla in these insects [29, 30]. Currently, only Li et al. [40] have studied on the ultrastructure of the antennal sensilla in the erotylid beetle *Dacne picta* Crotch (Erotylidae), and we further compared these sensilla found on the antennae of *T*. *ainonia* with the antennal sensilla of the erotylid beetle *D*. *picta*. It was found that the sensillum types of the two species are similar, except that *D*. *picta* have sensilla campaniform on the 9th flagellomere (Table 3).

**Table 3:** The sensillum types and morphological characteristics of *T*. *ainonia* and *D*. *picta* antennae.

Types of antennal sensilla		Morphology	
		*T*. *ainonia*	*D*. *picta*
BB		Bristly, upright	Short thorn-like, upright
SC	SC 1	Thorn-like, with a socket at the base, the surface has longitudinal grooves, and forms an angle of less than 45° with antenna surface	Upright, slender, the surface has longitudinal grooves, with a socket at the base, and significantly higher than other sensilla
	SC 2	Bristled, grooved wall, and slightly curved to the outside of the antennal surface, with a socket at the base, with a pore at the top	Tilted, not bent, and forms an angle of 40–50° with the antenna surface, with a socket at the base
	SC 3	Similar to SC 2, with a disc-shaped pore at the top, and two small triangular shields on both sides of the socket, and significantly higher than other sensilla	Short, tilted, and forms an angle of 20–30° with the antenna surface, with a socket at the base
SB	SB 1	The double-walled basiconic sensillae, characterized by a short, fluted cuticular surface with a grooved peg-like appearance distally	Tilted, not bent, the base with an annular protuberance, and the top obtuse
	SB 2	Finger-like, slightly curved, the base with no socket, but bulge. The surface smooth without pores, and the top obtuse	Similar to SB 1, but the whole sensilla curved
	SB 3	The base enlarged, upright, the surface smooth, and no grooves or pores are observed, and the top mastoid-like and obtuse, and no sockets at the base	Slender, straight or curved, tapering from base to end, the base with an annular protuberance
	SB 4	–	Similar to SB 3, but the bending degree is larger and begins to bend from the middle
ST		Hair-like, without sockets at the base, the top curved and sharp. The surface smooth-wall, without obvious pores	Slender, slightly curved, gradually thinning from the middle to the end, with a socket at the base, and forms an angle of 30–40° with the antenna surface
SS		Styloconica structure that grows from the cuticle of the antennal bulge, with a wrinkled, upright middle, the surface smooth-wall, and the top obtuse and with pore	Hair-like, perpendicular to the surface, born on the round protuberance on the surface of the antennae
SCA		–	The shape is mamillary, and the base with a circular bulge

BB: Böhm’s bristles; ST: Sensilla trichodea; SC: Sensilla chaetica; SB: Sensilla basiconica; SS: Sensilla styloconica; SCA: Sensilla campaniform; “–” indicates that no sensilla are observed.

Böhm’s bristles were found on the antennae of most insect species. The Böhm’s bristles observed in the male and female adults of *T*. *ainonia* are highly similar to the others Coleoptera species, such as lady beetle *Hippodamia variegata* (Goeze) (Coccinellidae) [41], longhorned beetle *Aromia bungii* (Faldermann) (Cerambycidae) [37], and ground beetle *Platynus dorsalis* (Pontoppidan) (Carabidae) [42]. In *T*. *ainonia*, Böhm’s bristles are located at the joint between the scape and the pedicel, and have movable bases. These two features indicate that Böhm’s bristles of this beetle species are mechanical receptors that sense the position and movement of the antenna, and the mechanical function of this sensillum was also reported in other studies [19, 40, 43, 44].

Sensilla chaetica are the most widely distributed sensilla on both male and female antennae, that was similar in other Coleoptera species [36, 45, 46]. The morphology of sensilla chaetica (including three subtypes, SC 1, SC 2, and SC 3) of *T*. *ainonia* are identical in shape to that found in other Coleoptera species [16, 46–48]. For example, the external morphology of SC 1 on the antennae of *T*. *ainonia* are similar to SC of *H*. *variegata* (Coccinellidae) [41], *P*. *dorsalis* (Carabidae) [42], and carabid beetle *Carabus elysii* Thomson (Carabidae) [48]. There are longitudinal grooves on the surface of SC 1, and the top of these sensilla is sharp without pores, indicating that they may be mechanical receptors [41, 48].

The external morphology and distribution of the SC 2 on the antennae of *T*. *ainonia* are similar to those of SC of *A*. *bungii* (Cerambycidae) [37], leaf beetle *Ophraella communa* Lesage (Chrysomelidae) [46], *C*. *elysii* (Carabidae) [48], and *H*. *variegata* (Coccinellidae) [41]. The SC 2 of *T*. *ainonia* are thicker than the SC 1, and distributed at the junction between the flagellomere, indicating that they are proprioceptors for detecting the position of the antennal segment [37]. In addition, the terminal end of these sensilla has a pore, suggesting that the sensilla also have chemical perception function [19].

The SC 3 identified on the antennae of *T*. *ainonia* is a long SC, which are characterized by being significantly longer than other types of sensilla. This character makes easier physical contact with the external environment, indicating a mechanical stimulation function [49]. However, the triangular shield at the base of SC 3 of *T*. *ainonia* limits the movement of the sensilla in the base direction, which suggest that the mechanical function of SC 3 is limited. Both SC 2, SC 3 has a pore at their tips, which indicates both gustatory and olfactory functions for these sensillum types [11, 21]. Therefore, the SC 1 on the antennae of *T*. *ainonia* are mechanoreceptor, and the SC 2 and SC 3 are most likely dual-functional receptors with mechanical perception and chemical perception functions [9, 37, 39, 49, 50].

In many insects, sensilla basiconica are common chemoreceptor [19, 51–53]. The sensilla basiconica of *T*. *ainonia* have three subtypes (SB 1, SB 2, and SB 3). The morphology of SB 1 is similar to that of granary weevil *Sitophilus granaries* (Linnaeus) (Curculionidae) [54], bruchid beetle *Callosobruchus maculatus* (Fabricius) (Bruchidae), seed beetle *Callosobruchus rhodesianus* (Pic) (Bruchidae), and seed beetle *Callosobruchus subinnotatus* (Pic) (Bruchidae) [55, 56]. These sensilla were called double-walled basiconic sensilla that characterized by a short, fluted cuticular surface, with a grooved peg-like appearance distally [54]. Based on earlier reports, this sensillum type may be thermo- or hygroreceptors, or both [55].

The SB 2 on the antennae of *T*. *ainonia* are highly consistent with the SB 1 identified by Li et al. on the antennae of *D*. *picta* (Erotylidae) [40]. In addition, the external morphology of SB 2 is similar to the SB of *O*. *communa* (Chrysomelidae) and longhorn beetle *Phoracantha semipunctata* Fabricius (Cerambycidae) [46, 57]. It characterized by no sockets at the base, and the surface of these sensilla of *T*. *ainonia* is porous. These morphological features indicate that the sensilla mainly had chemical perception function. Previous studies have also shown that porous sensilla basiconica have olfactory function [9, 57]. It is worth noting that in the antennae of *T*. *ainonia*. SB 2 are abundantly distributed on the F7–F9 of the flagellum, and in the F7 and F8 segments, these sensilla are only distributed near the junction of the two segments, so they are speculated that the sensilla play a buffering role in antennal activity.

The SB 3 on the antennae of *T*. *ainonia* are characterized by a smooth wall without longitudinal grooves, a uniform thickness, and a distinctive droplet shape at the tip, which are similar to the sensilla basiconica of the *H*. *variegata* (Coccinellidae) [41] and cigarette beetle *Lasioderma serricorne* (Fabricius) (Anobiidae) [10]. These sensilla are distributed in a few numbers on the 9th flagellomere of the antennae of *T*. *ainonia*. The exact function of these sensilla is not clear, but may be related to the reception of pheromone and plant volatile.

The external morphology and distribution of sensilla trichodea (ST) found on the antennae of *T*. *ainonia* are highly similar to ST of *A*. *bungii* (Cerambycidae) [37], *H*. *variegata* (Coccinellidae) [41], harlequin ladybird *Harmonia axyridis* Pallas (Coccinellidae) [58], and ground beetle *Bembidion properans* Steph (Carabidae) [43]. The ST are slender, no obvious pores were observed on these sensilla. The sensilla are densely distributed on the tip of flagellum. Previous studies suggested that ST act as both mechanical or tactile sensilla [18, 59], while other studies suggested that ST might have olfactory functions in detecting sex pheromones [42, 59].

Sensilla styloconica (SS) are common in Lepidoptera [23–25], and also found in Hemiptera, Hymenoptera, Diptera, and Coleoptera [27, 60–63]. There are folds in the middle of SS, which make this type of sensilla has a double-layer structure, and there is an obvious pore at its tip, that indicate olfactory function. Sensilla styloconica have been shown to have the thermo-hygroreceptive and olfactory function in some Coleoptera species [17, 18, 64]. In addition, SS were significantly more abundant in females than males of *T*. *ainonia*. It is speculated that it may be due to the fact that *T*. *ainonia* likes to live in a dark and humid environment, and a large number of the SS on the antennae of females is conducive to the selection of oviposition sites by females.

In conclusion, five types of antennal sensilla and their morphology, number, and distribution for both sexes of *T*. *ainonia* were identified. Some sensilla showed sexually dimorphic. The number of the SC 1 on the antennae of males was significantly higher than that of females. According to the potential functions of these sensilla, the sexually dimorphic may be related to the males’ active search for mating. In addition, the number of the SS on the antennae of females was significantly higher than that of males. This may be related to the reproduction of the female, because the eggs need to be in a humid environment to complete the development. Our results provide a morphological basis for further study on the host localization, mating, and other behavior of the fungal pest *T*. *ainonia*. Further anatomical and electrophysiological studies are needed to confirm the function of the antennal sensilla of *T*. *ainonia*.
